# Changes in the estimated glucose disposal rate and incident cardiovascular disease in patients with cardiovascular–kidney–metabolic syndrome stages 0–3: a prospective cohort study in China

**DOI:** 10.1186/s12872-025-05300-8

**Published:** 2025-11-26

**Authors:** Jia Liu, Wendong Xu, Yue Zhang, Yuan Jia, Shiru Bai, Lu Er, Rongpin Du

**Affiliations:** Department of Cardiology, Hebei General Hospital, Heping West Road, Shijiazhuang, 050000 NO. 348Hebei China

**Keywords:** Cardiovascular disease, Cardiovascular–kidney–metabolic syndrome, Insulin resistance, Estimated glucose disposal rate, CHARLS, Prospective cohort study

## Abstract

**Background and aims:**

Cardiovascular–kidney–metabolic syndrome (CKM) significantly increases the burden of cardiovascular disease (CVD), particularly in China, which has a rapidly aging population. The estimated glucose disposal rate (eGDR) is a reliable indicator for assessing insulin resistance (IR), but its dynamic changes and association with the risk of new-onset CVD in patients with CKM syndrome have not been fully elucidated. The aim of this study was to investigate the associations between dynamic changes in and cumulative of eGDR (cumeGDR) and the risk of new-onset CVD in Chinese adults with CKM syndrome.

**Methods:**

A total of 2862 patients with CKM syndrome stages 0–3 without CVD at baseline from the China Health and Retirement Longitudinal Study (CHARLS) were enrolled. K-means clustering was used to measure the change in eGDR from 2012 to 2015, and the cumulative eGDR level was calculated. Logistic regression, restricted cubic splines (RCS), and subgroup analysis were used to explore the potential associations between changes in the eGDR and the risk of new-onset CVD (including heart disease and stroke) in patients with CKM syndrome stages 0–3.

**Results:**

During the 3-year follow-up period, 404 (14.1%) CVD events occurred, including 254 heart disease cases and 177 stroke cases. After adjusting for confounding factors, compared with the group with persistently high eGDR level (Class 1), the groups with significantly decreased eGDR level (Class 2) and persistently low eGDR level (Class 3) had a significantly increased CVD risk (Class 2: OR = 1.82 [1.36–2.45], *P* < 0.001;Class 3: OR = 1.90 [1.41–2.56], *P* < 0.001). Further RCS regression analysis revealed a negative linear association between the cumulative eGDR and CVD risk(P for overall < 0.001, nonlinear *P* = 0.922).

**Conclusion:**

Persistently low eGDR level are associated with an increased risk of new-onset CVD in those with CKM syndrome stages 0–3. Continuous dynamic monitoring of the eGDR may help identify high-risk individuals with CKM syndrome stages 0–3 and provide critical evidence for early intervention.

**Supplementary Information:**

The online version contains supplementary material available at 10.1186/s12872-025-05300-8.

## Introduction

Cardiovascular–kidney–metabolic syndrome (CKM) was formally defined by the American Heart Association (AHA) in 2023. It is a systemic disease characterized by the pathophysiological interactions among obesity, diabetes, chronic kidney disease (CKD), and cardiovascular disease [1]. This pathophysiological interaction significantly increases the risk of serious adverse cardiac, renal, and metabolic events [2], leading to high incidences of morbidity, mortality, and health care burdens worldwide. Approximately 90% of adults worldwide have stage 1 or higher CKM syndrome [1, 3]. This widespread distribution of a high-risk population means that effective early intervention strategies have significant public health value. In China, this challenge is particularly acute, where CVD accounts for more than 45% of all-cause mortality, and the clinical burden of CKM syndrome is driven mainly by CVD [4]. Individuals with CKM syndrome stages 0–3 are often asymptomatic but require focused prevention of CVD events; however, traditional models often fail to comprehensively integrate metabolic, renal, and vascular biomarkers, which exacerbates the difficulty of early identification and precise risk stratification [1, 5].

Insulin resistance is a core driver of CKM syndrome progression [6], exacerbating metabolic dysregulation [7], endothelial damage [8], and organ fibrosis [9, 10]. The hyperinsulinemic–euglycemic clamp (HIEC) technique is the gold standard for detecting IR; however, the invasiveness, complexity, time-consuming nature, and high cost of this technique make it impractical for routine clinical practice and widespread application [11]. Homeostasis model assessment of insulin resistance (HOMA-IR), a surrogate marker for IR, is calculated on the basis of fasting insulin values, which are not commonly tested in routine clinical practice [12]. Therefore, there is an urgent need to find alternative markers of IR that are easily accessible and applicable in routine clinical practice. Recently, the estimated glucose disposal rate (eGDR), which is calculated from readily available clinical parameters, including hemoglobin A1c (HbA1c), hypertension, and waist circumference (WC), has emerged as a more convenient surrogate marker for IR [13] and is more suitable for large-scale application and routine clinical practice. Compared with HOMA-IR, eGDR performs better in predicting cardiovascular risk, all-cause mortality, and cardiovascular mortality [14–16]. A higher eGDR indicates lower level of IR, whereas a lower eGDR indicates higher level of IR. eGDR has been shown in a recent study to identify those at high risk of CVD in patients with CKM stages 0–3 [4, 17].

However, a single eGDR measurement reflects a static IR status and fails to capture the dynamic changes in IR over time and their potential impact. Given the central driving role and possible time-varying nature of IR in CKM syndrome, systematically evaluating the relationship between the longitudinal trajectory of eGDR change and CVD risk is crucial for achieving more precise dynamic risk stratification and disease management. Unfortunately, there is an extreme paucity of research in this area.

Therefore, the aim of this study was to utilize prospective cohort data from the CHARLS to explore the associations between the dynamic trajectory of eGDR after baseline and the risk of new-onset CVD in Chinese adult patients with CKM syndrome. In addition, to assess the impact of long-term IR exposure, we also examined the association between cumulative eGDR level and the risk of new-onset CVD in Chinese patients with CKM syndrome.

## Methods

### Data source and study population

The data used in this study were derived from the China Health and Retirement Longitudinal Study, which is a nationally representative longitudinal cohort designed to investigate the health, socioeconomic, and behavioral characteristics of middle-aged and elderly individuals (≥ 45 years) in China. The CHARLS baseline survey was initiated in 2011 (Wave 1), covering 150 counties/districts and 450 villages/communities in 28 provinces across the country and utilizing a stratified multistage probability sampling methodology with a total of 17,708 respondents from 10,257 households, representing the diversity of the middle-aged and elderly population in China. Follow-up surveys were conducted every 2–3 years, including those in 2013 (Wave 2), 2015 (Wave 3), 2018 (Wave 4), and 2020 (Wave 5). In addition to questionnaires, CHARLS collected venous blood samples from participants in Wave 1 and Wave 3 to measure biomarkers, including glucose, lipids, and other biomarkers, which provide important data to support the study of metabolic diseases. Detailed information on sampling methods, anthropometric measurements and blood biomarker information for CHARLS has been previously documented in previous publications.

The datasets from Wave 1 (baseline, 2011) and Wave 3 (follow-up, 2015) were extracted, considering the available blood test data. Each participant was required to fast overnight before sample collection. Early the following morning, well-trained staff collected venous blood samples, which were immediately stored in a laboratory at a temperature below 4℃ after collection and were transferred to the Chinese Center for Disease Control and Prevention for centralized storage and subsequent analysis within two weeks. All research laboratories were standardized and accredited. The fasting blood glucose (FBG) concentration was measured via the enzyme colorimetric method, and glycated hemoglobin A1c (HbA1c) was evaluated uniformly at Youanmen Clinical Laboratory Center of Capital Medical University. This center adopted the standardized method of high-performance liquid chromatography (HPLC) to ensure high precision and batch-to-batch consistency in test results [18].

**Fig. 1:**
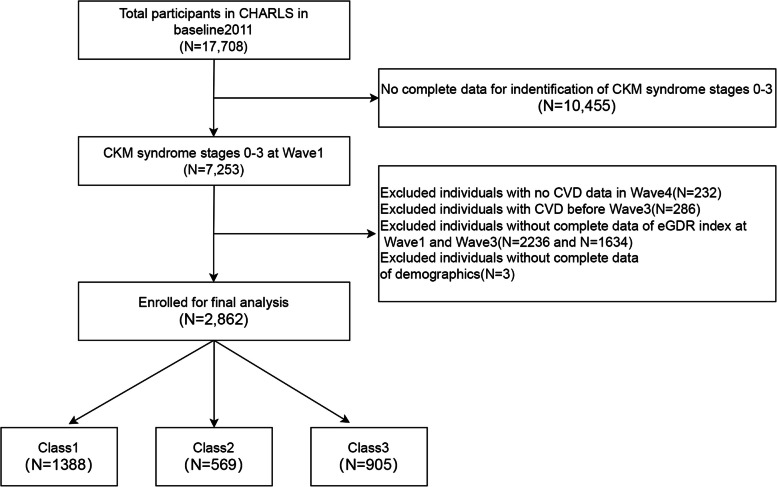
.

We first included 11,847 participants who completed blood tests in Wave 1 (2011), as blood samples were only collected in Waves 1 and 3. A total of 2,862 participants were included in the final study after meeting the following exclusion criteria: (1) inability to define the CKM syndrome stage in Wave 1, (2) lack of CVD status in subsequent follow-up, (3) inability to define eGDR in both Wave 1 and Wave 3, (4) lack of demographic information, and (5) preexisting CVD before 2015. Figure 1 presents the study population selection process.

The CHARLS study was approved by the Ethics Review Board of Peking University Biomedical (IRB00001052–11015). All participants signed written informed consent forms before the start of the study. The principles outlined in the Strengthening the Reporting of Observational Studies in Epidemiology (STROBE) were followed in the current study [19]. Detailed information on the CHARLS data is accessible on its official website (http://charls.pku.edu.cn/en) [18].

### Data assessment and definitions

#### Assessment of exposure

The eGDR was computed utilizing the following formula:

$$\begin{aligned}\mathrm{eGDR}=21.158-\left(0.09\times\mathrm{WC}\right)-\left(3.407\times\mathrm{HT}\right)-\left(0.551\times\mathrm{HbA}1\mathrm c\right)\end{aligned}$$ [13], where WC is waist circumference (cm) and HT is hypertension status (yes = 1, no = 0), HbA1c denoting the percentage of HbA1c.

The cumulative of eGDR was calculated as follows:

$$\begin{aligned}\mathrm{CumeGDR}&=\left[({\mathrm{eGDR}}_{2012}+{\mathrm{eGDR}}_{2015})/2\right]\\&\ast\mathrm{time}\left(2015-2012\right)\end{aligned}$$

To ensure the comparability and standardization of eGDR measurements at different time points and research sites, all data collection and detection processes in this study followed a uniform and rigorous standard operating procedure. All physical measurements were performed by uniformly trained researchers using standardized equipment to minimize human error [18]. Waist circumference (WC) was measured when the subject stood upright, using a soft tape measure placed horizontally at the level of the umbilicus. Measurements were taken at the end of a calm expiration while the subject held the breath, with an accuracy of 0.1 cm.

#### Assessment of outcomes

The outcome of this study was the incidence of CVD, including heart disease and stroke. Consistent with previous studies [20–22], incident CVD events were assessed by standardized questions:"Have you been diagnosed by a doctor as having a stroke/heart disease (including a heart attack, coronary heart disease, angina pectoris, congestive heart failure, or other heart problems)?" or "Are you currently receiving any of the following treatments (taking traditional Chinese medicines/taking modern Western medicines/other treatments/none of the above) for stroke/heart disease or its complications?". Participants who reported "yes" to a physician diagnosis of heart disease or stroke or indicated receiving specific treatment for heart disease or stroke were defined as having cardiovascular disease. In addition, if participants reported a heart attack or stroke during the previous follow-up, they were required to validate the presence of CVD during the subsequent follow-up. If participants denied a previously self-reported diagnosis of heart attack or stroke, these inconsistencies were retrospectively corrected.

#### Definition of CKM syndrome stages 0 to 4

The stages of CKM syndrome from 0 to 4 are classified in accordance with the AHA Presidential Advisory Statement [1]. The CKM syndrome stages 0–4 are specified as follows: stage 0: no CKM syndrome health risk factors; stage 1: abdominal obesity and/or prediabetes; stage 2: metabolic disorders(type 2 diabetes, hypertension, and high triglycerides) or renal disorders; stage 3: subclinical CVD in the context of CKM syndrome; and stage 4: clinical CVD (coronary heart disease, heart failure, stroke, peripheral artery disease, atrial fibrillation) in the context of CKM syndrome. Subclinical CVD is defined as having a ≥ 20% 10-year CVD risk or high-risk CKD according to the American Heart Association (AHA) Predicting Risk of CVD Events (PREVENT) equations.

### Data collection

This study collected the following data: (1) demographic characteristics: gender, age, educational status (primary school or below, secondary school, college or above), hukou status (urban/rural), and marital status (married or other); (2) lifestyle factors: smoking status (never, former, current) and drinking status (never, former, current); (3) physical measurements: systolic blood pressure (SBP), diastolic blood pressure (DBP), and body mass index (BMI, kg/m^2^). Blood pressure measurements were performed at the examination center by trained medical staff using a standardized electronic sphygmomanometer (Omron HEM-7200). Following a period of quiet rest, systolic blood pressure (SBP) and diastolic blood pressure (DBP) were measured three times, and the average of these readings was recorded as the final value. (4) medical history: self-reported physician-diagnosed hypertension, diabetes, dyslipidemia, kidney disease, liver disease, lipid-lowering therapy, antihypertensive therapy, and diabetes treatment. Hypertension was defined as an average SBP ≥ 140 mmHg, or an average DBP ≥ 90 mmHg at baseline, or current use of antihypertensive medication, or self-reported history of hypertension [23]. Diabetes was defined as a fasting blood glucose level ≥ 126 mg/dl (7 mmol/L) and/or a random blood glucose level ≥ 200 mg/dl (11.1 mmol/L) and/or an HbA1c level ≥ 6.5% at baseline and/or self-reported history of diabetes or current use of anti-diabetic medication [24]. Other medical conditions were ascertained on the basis of self-reported history or receipt of any condition-specific treatment. (5) Laboratory examination: serum creatinine (Scr), blood urea nitrogen (BUN), uric acid (UA), estimated glomerular filtration rate (eGFR), fasting blood glucose (FBG), HbA1c, total cholesterol (TC), triglycerides (TG), high-density lipoprotein cholesterol (HDL-C), and low-density lipoprotein cholesterol (LDL-C) level. The eGFR was determined using the Chronic Kidney Disease Epidemiology Collaboration (CKD-EPI) equation [25].

### Handling of missing variables

Additional file 1: Table S1 illustrates the distribution of missing data for the included study participants. To maximize the sample size, we used multiple interpolation with chained equations (MICE) and implemented this multiple interpolation process through the “MICE” package, despite the small proportion of missing data.

### Statistical analysis

Normally distributed continuous variables are expressed as the means ± standard deviations (SDs), whereas nonnormally distributed variables are presented as the medians with interquartile ranges (IQRs). Categorical variables are reported as frequencies with percentages (%). Group comparisons were performed with χ^2^ tests for categorical variables, ANOVA for normally distributed continuous variables, and the Kruskal‒Wallis test for nonparametric data.

**Fig. 2:**
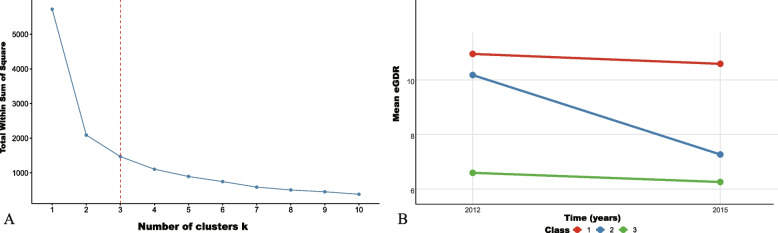
.

First, participants were classified into three groups using K-means clustering on the basis of their eGDR values from 2012 to 2015. K-means clustering is an unsupervised machine learning technique that aims to divide N observations into K clusters, which can effectively capture the subtle changes of dynamic eGDR. In order to determine the optimal number of clusters, we systematically evaluated scenarios from K = 2 to K = 10 and adopted the elbow rule as the primary criterion for judgment [26]. As shown in Fig. 2A, the reduction rate of within-cluster variance (distortion) declined significantly when K = 3, indicating that adding more clusters yields negligible explanatory power (the ‘elbow’ criterion). To ensure the repeatability of analyses, all analyses were implemented using the stats package in R software version 4.3.1 with a fixed random seed (seed = 123). All data preprocessing and clustering steps were integrated into an executable standardized workflow. To validate the robustness of the clustering results, we further calculated the silhouette coefficient and obtained an acceptable value (0.487), indicating that each cluster exhibits reasonable internal cohesion and inter-cluster separation at K = 3. Additionally, we assessed the stability of cluster assignment using Bootstrap resampling (n = 1000 iterations). Results revealed a high average consistency index of 0.974, with 98.1% of participants consistently assigned to the same cluster across repeated samples. To further confirm the validity of the clustering structure, we compared the K-means results with the hierarchical clustering method, which demonstrated high consistency (adjusted Rande index = 0.671; consistency index = 0.850). Although the elbow rule involves a degree of subjectivity, we determined the final number of clusters by combining statistical metrics and clinical interpretability, thereby effectively reducing the potential bias caused by subjective judgment. Accordingly, as shown in Fig. 2B, the participants were stratified into three classes. Class 1 was characterized by persistently high eGDR level, with eGDR ranging from 10.96 ± 1.38 in 2012 to 10.59 ± 0.81 in 2015, indicating optimal eGDR control. Class 2 was characterized by initially high eGDR that significantly decreased to low level, with eGDR ranging from 10.18 ± 1.28 in 2012 to 7.27 ± 1.11 in 2015, suggesting impaired eGDR control. Class 3 was characterized by persistently low eGDR level, with eGDR ranging from 6.59 ± 0.99 in 2012 to 6.26 ± 1.36 in 2015, indicating poor eGDR control. Additionally, we used continuous cumeGDR and quartiles of the cumeGDR group to assess the associations between long-term changes in the eGDR and CVD incidence.

Logistic regression analyses were conducted to evaluate the associations between cumeGDR, eGDR control level and CVD events, with odds ratios (ORs) and 95% confidence intervals (95% CIs) calculated across three models. The crude model was unadjusted for covariates. Model 1 was adjusted for age and gender. Model 2 included adjustments for all covariates included in Model 1 and in addition was adjusted for hukou status, education status, marital status, smoking status, drinking status, TC, LDL-C, eGFR, diabetes, lipid-lowering therapy, antihypertensive therapy, and diabetes treatment. To assess the incremental predictive value of dynamic changes in eGDR beyond traditional cardiovascular risk factors, we performed a comparative analysis utilizing nested logistic regression models. The basic model comprised established cardiovascular risk factors in the Chinese population: age, sex, smoking status, drinking status, TC, LDL-C, diabetes, and eGFR. The expanded model integrated eGDR dynamic change patterns into the basic model. Model performance was evaluated using the following indicators: (1) The Likelihood ratio test for comparing model fit; (2) The Akaike Information Criterion (AIC) for evaluating complexity-adjusted model improvement; (3) The Area under the receiver operating characteristic curve (AUC) for contrasting discriminative capacity; (4) The Net Reclassification Index (NRI) and Integrated Discrimination Improvement (IDI) for assessing advancements risk reclassification. Multicollinearity was tested using the variance inflation factor (VIF) method, with a VIF ≥ 5 indicating the presence of multicollinearity. Additional file 2: Table S2 indicating evidence of no significant multicollinearity. The area under the receiver operating characteristic curve (AUC) was used to evaluate the predictive ability of cumeGDR for CVD risk, and the “addfor” algorithm was employed to determine the optimal cutoff point. Furthermore, restricted cubic spline (RCS) regression analysis was employed to examine linear and dose‒response relationships between cumeGDR and CVD incidence in participants with CKM syndrome. Additionally, various subgroup and interaction analyses were performed to detect potential effect modifications. The participants were stratified into subgroups by age (< 60 years vs. ≥ 60 years), gender (male vs. female), marital status (married vs. other), hukou status (urban/rural), and education status (< middle school vs. ≥ middle school).

Sensitivity analysis were performed to assess the robustness of the primary results. First, we applied a logistic regression model that excluded participants with missing values for covariates to mitigate the potential influence of missing values on the primary results. Second, the data were reanalyzed after excluding participants with cancer at baseline (2015) to assess the potential impact of preexisting cancer on the observed associations. Third, we extended the follow-up of these participants until 2020 to test the stability of the results over a longer time period. Fourth, we excluded participants who experienced cardiovascular events during early follow-up (2018) and repeated the analysis. Finally, to minimize reverse causality, we excluded participants in CKM stage 3 at baseline those with existing subclinical CVD from the cohort, retaining only those in CKM stages 0**–**2, and repeated the analysis.

All the statistical analyses were performed using R software version 4.3.1 (http://www.R-project.org/), and a two-sided *P* < 0.05 was considered statistically significant.

## Results

### Population characteristics

Figure 1 illustrates the inclusion and exclusion criteria used in this study. Specifically, we included 17,708 participants in Wave 1 of the CHARLS cohort. Of these, 10,455 participants were unable to be classified into CKM syndrome stage in Wave 1, 232 participants were excluded because of incomplete follow-up data, 286 participants were excluded because they had CVD at baseline, 3,870 participants were excluded because of missing eGDR data in Waves 1 and 3, and 3 participants were excluded because of a lack of demographic data. A total of 2,862 participants with a mean age of 57 years (51–63 years) were included in the final study, of which 1,563 (54.6%) were female. The average eGDR of the participants was 9.42 in 2012 and 8.56 in 2015. The cumeGDR for the entire cohort was 28.22. Table 1 summarizes the baseline characteristics of participants with CKM syndrome stages 0–3 in the three groups on the basis of the level of eGDR control (Classes 1–3). These classes were significantly different in terms of age, gender, education level, smoking, drinking, CKM syndrome stage, BMI, SBP, DBP, comorbidities (hypertension, diabetes, dyslipidemia), medication status (antihypertensive drugs, hypoglycemic drugs, lipid-lowering drugs), and biochemical markers (HbA1c, FBG, TG, TC, HDL-C, LDL-C, UA, eGFR). Specifically, Class 3 represents the most severely metabolically dysregulated patients, with an older age, higher BMI and blood pressure, more comorbidities, and more severe biochemical abnormalities than the other groups. Table 1 summarizes the baseline characteristics of the participants with stages 0–3 CKM syndrome in the 3 groups on the basis of the level of eGDR control (Classes 1–3). In addition, baseline characteristics stratified by cumeGDR quartiles are detailed in Table S3.

**Table 1:** Baseline characteristics according to eGDR by K-means clustering analysis in CKM syndrome stages 0–3

Characteristic	Overall (*N* = 2862)	Class1 (*N* = 1388)	Class2 (*N* = 569)	Class3 (*N* = 905)	*P* value
Age,years	57.00 [51.00, 63.00]	55.00 [49.00, 61.00]	58.00 [52.00, 65.00]	59.00 [53.00, 65.00]	< 0.001
Gender,%					0.038
Female	1299 (45.4)	619 (44.6)	285 (50.1)	395 (43.6)	
Male	1563 (54.6)	769 (55.4)	284 (49.9)	510 (56.4)	
Marital status,%					0.336
Married	2509 (87.7)	1229 (88.5)	491 (86.3)	789 (87.2)	
Others	353 (12.3)	159 (11.5)	78 (13.7)	116 (12.8)	
Education,%					0.001
College or above	73 (2.6)	32 (2.3)	16 (2.8)	25 (2.8)	
Elementary school or below	1922 (67.2)	882 (63.5)	399 (70.1)	641 (70.8)	
Middle school	867 (30.3)	474 (34.1)	154 (27.1)	239 (26.4)	
Hukou status,%					0.071
Agriculture	2462 (86.0)	1215 (87.5)	484 (85.1)	763 (84.3)	
Others	400 (14.0)	173 (12.5)	85 (14.9)	142 (15.7)	
Smoking status					0.001
Current Smoker	872 (30.5)	422 (30.4)	203 (35.7)	247 (27.3)	
Former Smoker	204 (7.1)	81 (5.8)	44 (7.7)	79 (8.7)	
Never Smoked	1786 (62.4)	885 (63.8)	322 (56.6)	579 (64.0)	
Drinking status					< 0.001
Current Drinker	895 (31.3)	418 (30.1)	198 (34.8)	279 (30.8)	
Former Drinker	190 (6.6)	70 (5.0)	35 (6.2)	85 (9.4)	
Never Drinker	1777 (62.1)	900 (64.8)	336 (59.1)	541 (59.8)	
CKM syndrome stages, %					< 0.001
0	114 (4.0)	108 (7.8)	6 (1.1)	0 (0.0)	
1	356 (12.4)	302 (21.8)	54 (9.5)	0 (0.0)	
2	833 (29.1)	345 (24.9)	175 (30.8)	313 (34.6)	
3	1559 (54.5)	633 (45.6)	334 (58.7)	592 (65.4)	
BMI,kg/m^2^	23.28 [21.09, 25.80]	22.16 [20.39, 24.14]	23.61 [20.93, 26.41]	25.23 [23.00, 27.52]	< 0.001
SBP,mmHg	125.33 [114.00, 140.33]	116.33 [108.33, 125.67]	126.67 [118.33, 135.67]	145.00 [133.33, 156.67]	< 0.001
DBP,mmHg	74.33 [67.33, 82.33]	69.67 [63.67, 75.67]	74.33 [68.00, 80.67]	83.33 [75.33, 90.67]	< 0.001
Diabetes,%					< 0.001
No	2702 (94.4)	1361 (98.1)	534 (93.8)	807 (89.2)	
Yes	160 (5.6)	27 (1.9)	35 (6.2)	98 (10.8)	
Hypertension,%					< 0.001
No	1810 (63.2)	1303 (93.9)	480 (84.4)	27 (3.0)	
Yes	1052 (36.8)	85 (6.1)	89 (15.6)	878 (97.0)	
Dyslipidemia,%					< 0.001
No	2627 (91.8)	1329 (95.7)	534 (93.8)	764 (84.4)	
Yes	235 (8.2)	59 (4.3)	35 (6.2)	141 (15.6)	
Kidney disease,%					0.051
No	2770 (96.8)	1332 (96.0)	554 (97.4)	884 (97.7)	
Yes	92 (3.2)	56 (4.0)	15 (2.6)	21 (2.3)	
Liver disease,%					0.323
No	2787 (97.4)	1354 (97.6)	549 (96.5)	884 (97.7)	
Yes	75 (2.6)	34 (2.4)	20 (3.5)	21 (2.3)	
Lipid-lowering treatment,%					< 0.001
No	2747 (96.0)	1362 (98.1)	553 (97.2)	832 (91.9)	
Yes	115 (4.0)	26 (1.9)	16 (2.8)	73 (8.1)	
Antihypertensive treatment,%					< 0.001
No	2431 (84.9)	1385 (99.8)	540 (94.9)	506 (55.9)	
Yes	431 (15.1)	3 (0.2)	29 (5.1)	399 (44.1)	
Hypoglycemic treatment,%					< 0.001
No	2760 (96.4)	1371 (98.8)	546 (96.0)	843 (93.1)	
Yes	102 (3.6)	17 (1.2)	23 (4.0)	62 (6.9)	
HbA1c, %	5.10 [4.90, 5.40]	5.10 [4.80, 5.30]	5.20 [4.90, 5.50]	5.20 [5.00, 5.60]	< 0.001
FBG, mg/dL	102.60 [95.22, 112.32]	100.44 [93.42, 108.54]	103.86 [95.58, 115.56]	104.94 [98.28, 118.80]	< 0.001
TG, mg/dL	105.32 [74.34, 155.76]	95.58 [69.03, 134.52]	101.78 [71.68, 154.88]	126.56 [87.61, 184.08]	< 0.001
TC, mg/dL	191.37 [168.17, 216.11]	185.57 [164.30, 209.25]	191.37 [169.33, 218.43]	198.33 [175.13, 224.61]	< 0.001
HDL-c, mg/dL	49.10 [40.59, 59.92]	51.42 [42.53, 61.86]	49.48 [39.43, 61.47]	46.39 [38.27, 55.28]	< 0.001
LDL-c, mg/dL	115.21 [94.33, 139.18]	111.92 [92.78, 134.15]	115.98 [92.40, 140.34]	120.62 [97.81, 144.98]	< 0.001
BUN,mg/dL	14.96 [12.50, 17.93]	14.97 [12.58, 17.79]	14.90 [12.38, 18.01]	15.01 [12.52, 18.15]	0.988
Creatinine,mg/dL	0.73 [0.64, 0.86]	0.75 [0.63, 0.85]	0.73 [0.64, 0.86]	0.73 [0.66, 0.88]	0.296
Uric acid, mg/dL	4.17 [3.46, 5.01]	4.02 [3.35, 4.78]	4.24 [3.48, 5.04]	4.38 [3.64, 5.31]	< 0.001
eGFR, mL/min/1.73m^2^	96.51 [86.87, 103.80]	98.17 [87.49, 104.77]	96.38 [87.60, 103.87]	94.37 [84.93, 101.35]	< 0.001
Cumulative eGDR	28.22 [21.64, 32.26]	32.32 [30.62, 33.95]	26.18 [24.52, 27.78]	19.52 [17.49, 21.43]	< 0.001
CVD					< 0.001
No	2458 (85.9)	1264 (91.1)	476 (83.7)	718 (79.3)	
Yes	404 (14.1)	124 (8.9)	93 (16.3)	187 (20.7)	
Heart disease,%					< 0.001
No	2608 (91.1)	1308 (94.2)	504 (88.6)	796 (88.0)	
Yes	254 (8.9)	80 (5.8)	65 (11.4)	109 (12.0)	
Stroke,%					< 0.001
No	2685 (93.8)	1340 (96.5)	532 (93.5)	813 (89.8)	
Yes	177 (6.2)	48 (3.5)	37 (6.5)	92 (10.2)	

*BMI* Body Mass Index, *SBP* Systolic blood pressure, *DBP* Diastolic blood pressure, *HbA1c* Glycated hemoglobin A1c, *FBG* Fasting blood glucose, *TG* Triglycerides, *TC* Total cholesterol, *HDL-c* High-density lipoprotein cholesterol, *LDL-c* Low-density lipoprotein cholesterol, *BUN* Blood urea nitrogen, *eGFR* Estimated glomerular filtration rate, *eGDR* Estimated glucose disposal rate, *CVD* Cardiovascular diseases

### Associations between eGDR changes and new-onset CVD

During the 3-year follow-up (from Wave 3 in 2015 to Wave 4 in 2018), 404 participants (14.1%) had CVD events, namely, 254 had heart attacks (8.9%) and 177 had strokes (6.2%). Logistic analysis was applied to study the relationship between eGDR changes and new-onset CVD. As shown in Table 2, different IR control states reflected by eGDR were observed after individuals were grouped via K-means clustering analysis. Compared with Class 1, Classes 2 and 3 had a significantly greater risk of new-onset CVD (Class 2: OR 1.82, 95% CI 1.36–2.45, *P* < 0.001; Class 3: OR 1.90, 95% CI 1.41–2.56, *P* < 0.001), heart disease (Class 2: OR 2.01, 95% CI 1.42–2.86, *P* < 0.001; Class 3: OR 1.78, 95% CI 1.23–2.55), *P* = 0.002, and stroke (Class 2: OR 1.67, 95% CI 1.06–2.61, *P* = 0.025; Class 3: OR 1.95, 95% CI 1.26–3.02, *P* = 0.003). Cumulative eGDR was also used to assess eGDR changes. When the first quartile (the group with the lowest cumulative level) was used as a reference, with increasing cumeGDR, the event rates of new CVD, heart disease, and stroke gradually decreased (*p* for trend < 0.001). These results remained consistent after correction for potential confounders. The AUC (95% confidence interval) for cumeGDR was 0.624. We found that CVD risk began to increase significantly when cumeGDR was lower than 7.97. We recommend taking this value as a potential cutoff point for identifying high-risk individuals requiring enhanced intervention.

**Table 2:** Logistic analysis for the association between different eGDR change and CVD

eGDR	Crude model	*P* value	Model1	*P* value	Model2	*P* value
	OR(95%CI)		OR(95%CI)		OR (95%CI)	
Change in the eGDR						
1	Ref		Ref		Ref	
2	1.99 (1.49–2.66)	< 0.001	1.90 (1.42–2.55)	< 0.001	1.82 (1.36–2.45)	< 0.001
3	2.65 (2.08–3.40)	< 0.001	2.43 (1.90–3.12)	< 0.001	1.90 (1.41–2.56)	< 0.001
Cumulative eGDR						
Q1	Ref		Ref		Ref	
Q2	0.60 (0.46–0.79)	< 0.001	0.62 (0.47–0.81)	< 0.001	0.75 (0.56–1.01)	0.063
Q3	0.40 (0.30–0.54)	< 0.001	0.44 (0.33–0.59)	< 0.001	0.56 (0.40–0.79)	< 0.001
Q4	0.33 (0.24–0.45)	< 0.001	0.36 (0.26–0.49)	< 0.001	0.47 (0.32–0.67)	< 0.001
p for trend		< 0.001		< 0.001		< 0.001
Per SD	0.65 (0.58–0.72)	< 0.001	0.67 (0.60–0.74)	< 0.001	0.74 (0.65–0.85)	< 0.001
Heart disease						
Change in the eGDR						
1	Ref		Ref		Ref	
2	2.11 (1.49–2.97)	< 0.001	2.06 (1.46–2.92)	< 0.001	2.01 (1.42–2.86)	< 0.001
3	2.24 (1.66–3.03)	< 0.001	2.07 (1.52–2.82)	< 0.001	1.78 (1.23–2.55)	0.002
Cumulative eGDR						
Q1	Ref		Ref		Ref	
Q2	0.74 (0.53–1.02)	0.07	0.78 (0.56–1.08)	0.138	0.89 (0.62–1.29)	0.546
Q3	0.47 (0.32–0.68)	< 0.001	0.51 (0.35–0.74)	< 0.001	0.60 (0.39–0.91)	0.018
Q4	0.43 (0.29–0.62)	< 0.001	0.47 (0.32–0.68)	< 0.001	0.56 (0.36–0.86)	0.009
p for trend		< 0.001		< 0.001		0.002
Per SD	0.71 (0.63–0.81)	< 0.001	0.74 (0.65–0.84)	< 0.001	0.79 (0.67–0.92)	0.002
Stroke						
Change in the eGDR						
1	Ref		Ref		Ref	
2	1.94 (1.24–3.01)	0.003	1.81 (1.16–2.81)	0.009	1.67 (1.06–2.61)	0.025
3	3.16 (2.22–4.56)	< 0.001	2.89 (2.02–4.19)	< 0.001	1.95 (1.26–3.02)	0.003
Cumulative eGDR						
Q1	Ref		Ref		Ref	
Q2	0.57 (0.39–0.82)	0.003	0.57 (0.39–0.83)	0.003	0.75 (0.49–1.15)	0.186
Q3	0.35 (0.23–0.54)	< 0.001	0.38 (0.24–0.59)	< 0.001	0.55 (0.33–0.91)	0.022
Q4	0.24 (0.15–0.39)	< 0.001	0.26 (0.16–0.42)	< 0.001	0.40 (0.22–0.69)	0.001
P for trend		< 0.001		< 0.001		< 0.001
Per SD	0.59 (0.51–0.69)	< 0.001	0.61 (0.52–0.71)	< 0.001	0.73 (0.60–0.88)	< 0.001

Crude model, unadjusted for covariates; Model 1, adjusted for age and gender; Model 2, adjusted for age, gender, hukou status, education status, marital status,smoking status, drinking status, TC, LDL-c, eGFR,diabetes, lipid-lowering therapy, antihypertensive therapy, and diabetes treatment

*eGDR* Estimated glucose disposal rate, *CVD* Cardiovascular diseases, *Q1* Quartile 1, *Q2* Quartile 2, *Q3* Quartile 3, *Q4* Quartile 4, *OR* Odds ratio, *CI* Confidence interval, *SD* Standard deviation

**Fig. 3:**
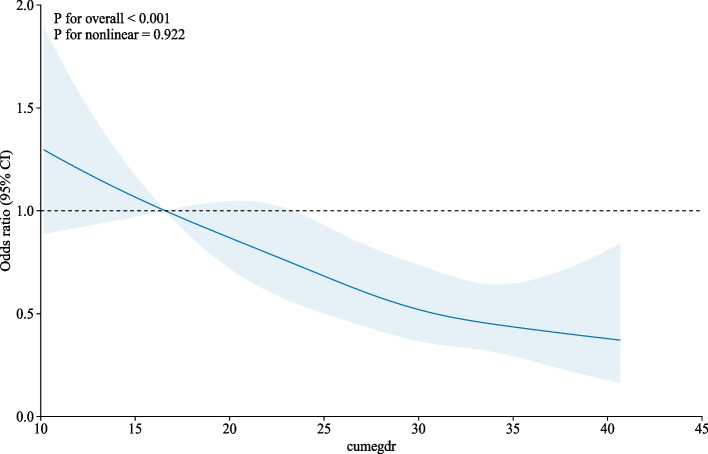
.

**Fig. 4:**
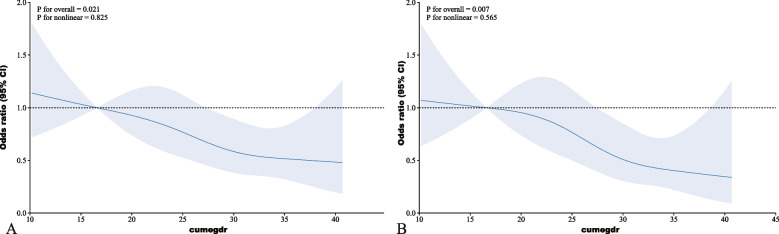
.

Restricted cubic spline (RCS) models were applied to assess the dose‒response relationships between cumeGDR and new-onset CVD, heart disease, and stroke. As shown in Fig. 3, the fully adjusted RCS model revealed a negative linear correlation between cumeGDR and new-onset CVD (*P* for overall < 0.001, P for nonlinearity = 0.922). In addition, as shown in Fig. 4, the RCS model revealed a negative linear correlation between cumeGDR and incident heart disease and stroke (heart disease: P for overall = 0.021, P for nonlinearity = 0.825; stroke: P for overall = 0.007, P for nonlinearity = 0.565).

### Incremental predictive value of eGDR dynamic changes for CVD risk

After adjusting for traditional cardiovascular risk factors, the dynamic change in eGDR remained significantly associated with the risk of CVD. Comparative analysis of the models revealed that the goodness of fit of the model was significantly improved after the addition of eGDR (likelihood ratio test: χ^2^ = 38.54, *P* < 0.001), with a decrease of 34.54 in the Akaike Information Criterion (AIC), indicating that the expanded model is superior when accounting for model complexity. Regarding model discriminative ability, the basic model predicted CVD with an AUC of 0.636 (95% CI: 0.607–0.664). After incorporating the dynamic changes in eGDR, the AUC increased to 0.660 (95% CI: 0.632–0.688), representing an improvement of 0.024 (*P* = 0.019). Furthermore, risk reclassification analysis revealed an NRI of 0.4119 and an IDI of 0.0143. These findings consistently indicate that incorporating the dynamic changes of eGDR into conventional risk models can significantly enhance the accuracy of CVD risk stratification in patients with CKM syndrome stages 0**–**3.

### Subgroup analysis

**Fig. 5:**
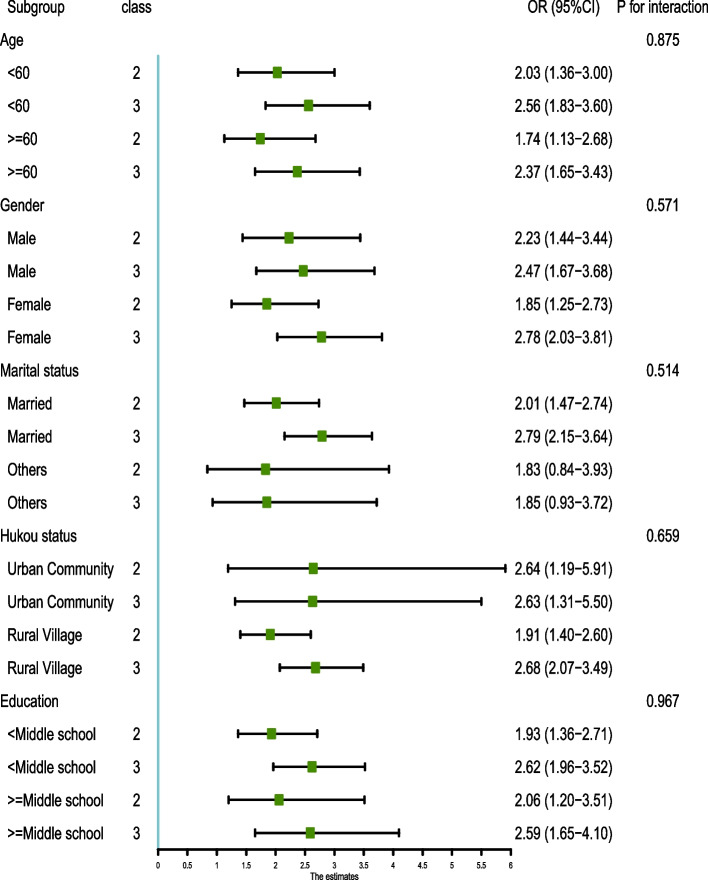
.

**Fig. 6:**
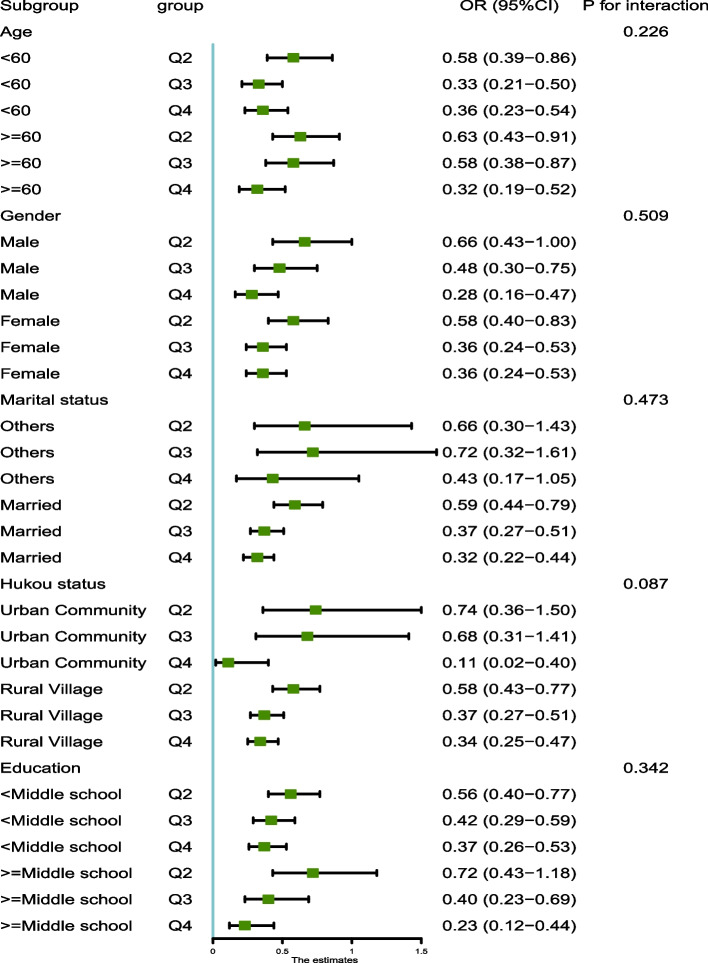
.

To further investigate the relationships between eGDR changes and cumeGDR and new-onset CVD, a series of subgroup analyses were conducted. As shown in Figs. 5 and 6, subgroups stratified by age, gender, hukou status, education, and marital status did not influence the relationships between eGDR changes and cumeGDR and new-onset CVD (all *P* > 0.05 for interaction).

### Sensitivity analysis

Sensitivity analyses confirmed the robustness of the primary results. First, after removing participants with missing covariates in the fully adjusted model, the association between the control level of eGDR and the incidence of CVD remained consistent with our study results (Class 2: OR 1.82, 95% CI 1.38–2.49, *P* < 0.001; Class 3: OR 1.95, 95% CI 1.44–2.62, *P* < 0.001). Furthermore, a negative correlation persisted between cumulative eGDR and the new-onset of CVD (*p* for trend < 0.001), as detailed in Additional File 4: Table S4. Second, after excluding individuals with cancer at the baseline in 2015, poor eGDR control level was associated with an increased risk of new-onset CVD (Class 2: OR 1.83, 95% CI 1.36–2.45, *P* < 0.001; Class 3: OR 1.92, 95% CI 1.42–2.57, *P* < 0.001) and the association between cumeGDR and CVD was consistent with the primary findings (*p* for trend < 0.001) (Additional file 4: Table S5). Third, we extended the follow-up of these participants until 2020, and consistent with our study, the risk of CVD continued to increase in the group with a significant decrease in eGDR and in the group with persistently low level (Class 2: OR 2.06, 95% CI 1.56–2.72, *P* < 0.001; Class 3: OR 2.31, 95% CI 1.75–3.04, *P* < 0.001). There was a negative correlation between cumeGDR and the incidence of CVD (*p* for trend < 0.001)(Additional file 4: Table S6). Fourth, after excluding participants who experienced cardiovascular events in the early follow-up period, the relationship between eGDR control level and CVD incidence remained consistent with our findings (Class 2: OR 1.79, 95% CI 1.15–2.75, *P* = 0.008; Class 3: OR 2.57, 95% CI 1.71–3.86, *P* < 0.001). There was a negative correlation between cumeGDR and the incidence of CVD (*p* for trend < 0.001)(Additional file 4: Table S7). Finally, excluding participants already in CKM syndrome stage 3 those with existing subclinical CVD confirmed the robustness of the initial findings: individuals with persistently low or markedly decreased eGDR levels still exhibited significantly higher risks of clinical CVD events (Class 2: OR 1.78, 95% CI 1.14–2.75, *P* = 0.01; Class 3: OR 2.03, 95% CI 1.27–3.21, *P* = 0.003). Furthermore, the negative linear association between cumeGDR and the risk of new-onset CVD remained robust (*p* for trend = 0.003) (Additional file 4: Table S8).

## Discussion

In this study, we dynamically assessed the longitudinal trajectory of eGDR in patients with CKM syndrome stages 0–3 by using K-means clustering analysis, revealing a significant association with cardiovascular disease risk. Unlike most previous cross-sectional studies or those relying on baseline single-point measurements, our analysis demonstrates that not only is the baseline state of insulin resistance important, but also its dynamic trajectory is a key determinant of prognosis. Specifically, we identified the “significantly decreased” and “persistently low” phenotypes as a high-risk population exhibiting sustained or progressively worsening metabolic dysfunction. This discovery elevates eGDR from a static marker of insulin resistance to a dynamic risk stratification tool, which is more capable of capturing the essence of CKM syndrome, a multisystem and progressive disease. Through a series of rigorous sensitivity analyses, we confirmed the robustness of the aforementioned association and effectively addressed concerns regarding potential reverse causality. To translate our findings into practical clinical tools, we further quantified the incremental predictive value of eGDR dynamic changes compared to traditional risk factors. This indicates that traditional risk prediction models may exhibit deficiencies in populations with CKM syndrome, while eGDR can partially address this shortcoming. Ultimately, the primary objective of all risk prediction models is to guide clinical practice. We identified that when cumeGDR falls below 7.97, CVD risk begins to increase significantly. This threshold can be regarded as the critical point for initiating or intensifying interventions. This value is highly consistent with the concept of “early, comprehensive, lifelong management” emphasized in CKM syndrome. Our research provides crucial evidence for the early identification of high-risk populations, holding significant clinical value for establishing dynamic risk prediction models and enabling precise interventions for high-risk individuals.

IR is characterized by reduced sensitivity to the physiological effects of insulin, which can lead to abnormal glucose and lipid metabolism, directly drive atherosclerosis, and induce endothelial dysfunction, chronic inflammation, and oxidative stress, accelerating arteriosclerosis and thrombosis [27]. In addition, IR is significantly associated with the risk of cardiovascular disease, chronic kidney disease, and metabolic disorders [7, 28, 29]. Particularly in the CKM syndrome population, IR drives cardiac, renal, and metabolic damage simultaneously, creating a vicious cycle [30] in which all three diseases often co-occur, and having more than one of these diseases at the same time exponentially increases the risk of death, primarily due to cardiovascular disease [31]; thus, early intervention reduces the risk of progression, and attention needs to be paid to evaluating IR. Although the hyperinsulinemic–euglycemic clamp (HIEG) and HOMA-IR are considered reliable metrics for assessing IR, their complex measurement techniques limit their practical application [11, 12]. Among alternative indicators, the TyG index demonstrates limited predictive stability due to its failure to integrate essential clinical parameters such as blood pressure and obesity. Recent studies have shown that in the popoulation of CKM syndrome stages 0**–**3, the TyG-BMI index combined with obesity parameters is closely associated with the future risk of CVD [32]. This not only validates the predictive role of insulin resistance in CKM syndrome patients but also highlights the importance of incorporating obesity parameters into IR assessment. To overcome the limitations of the above measurements, the eGDR was developed by the Pittsburgh Epidemiology of Diabetes Complications (EDC) study and further validated in type 1 diabetes [13]. The eGDR integrates metabolic, obesity, and glycemic control parameters and is superior to a single indicator, with a correlation of *r* = 0.63 with the HIEG clamp [33], and its predictive efficacy exceeds that of six alternative IR indicators (TyG index, TyG-waist circumference, TyG-body mass index, TyG-waist-to-height ratio, triglyceride-to-high density lipoprotein cholesterol ratio, and metabolic score for insulin resistance) [34, 35]. On the basis of these findings, eGDR seems to be a reasonable alternative indicator for IR estimation. The eGDR has been proven to effectively predict CVD events and stroke risk in different populations, including those with diabetes, those without diabetes, and the general population [15, 36]. Notably, in the CKM syndrome population, each 1-unit increase in eGDR was associated with a 9% reduction in the risk of CVD (HR 0.91, 95% CI 0.88–0.93) [4]. Given that patients in the early stages of CKM syndrome (stages 0–3) generally have visceral obesity and chronic inflammation and that IR is the core driving factor, this study focused on this population. A recent study in the Chinese population validated that the staging of CKM syndrome is significantly positively correlated with all-cause mortality [37], highlighting the importance of early risk stratification in this population. Regarding obesity and body fat distribution, abdominal obesity, high waist-to-height ratio and excessive obesity are prevalent among adults and are independently associated with important components of CKM syndrome such as hypertension, diabetes, and dyslipidemia [38]. These indicators are intrinsically linked to eGDR, as visceral fat accumulation exacerbates insulin resistance, thereby reducing eGDR. Consequently, the dynamic changes of eGDR may partially reflect the impact of visceral fat accumulation or alterations in metabolic activity, suggesting its synergistic predictive value alongside body composition changes. Incorporating obesity parameters into IR assessment is therefore essential. Previous studies have focused mostly on single-point measurements of baseline eGDR and have not considered the impact of eGDR fluctuations during follow-up. In contrast, the progression of CKM syndrome essentially reflects the continuous drift of metabolic parameters rather than a simple stage transition. Thus, capturing dynamic changes in the eGDR may be more capable of reflecting disease progression and risk than single-point measurements are. We applied K-means clustering to analyze the dynamic changes in eGDR and compared with the static limitation of the traditional quartile method. This method was used to identify three types of clinical phenotypes: persistently high, significantly decreased, and persistently low. A recent study in the general population revealed that participants with consistently low eGDR were associated with a significantly greater risk of CVD risk [39], confirming the importance of dynamic assessment. By identifying specific eGDR changes (such as persistently high, significantly decreased, and persistently low), it is possible to identify high-risk individuals more accurately, optimize risk stratification, and reflect the effectiveness of interventions through dynamic monitoring. For example, treatment with glucose-lowering drugs (such as glucagon-like peptide-1 receptor agonist, GLP-1RA) improves insulin sensitivity [40]. Previous studies have mostly assessed β-cell function by HOMA-IR, and in the future, we can use acquired metrics, such as eGDR, to provide a quantitative bridge for drug efficacy assessment. In addition to traditional drug therapies, nutritional intervention has also been proven to have the potential to improve metabolic parameters in CKM syndrome. For instance, certain dietary supplements (such as Flexovital) have shown efficacy in mitigating CKM-related indicators in animal models [41]. Although our study did not involve intervention trials, the dynamic changes of eGDR may serve as an intermediate indicator for evaluating the efficacy of nutritional intervention in future research.

Our study used K-means clustering analysis to divide CKM syndrome participants into three subgroups on the basis of their eGDR from 2012 to 2015. The results revealed that the eGDR in all subgroups tended to decrease, indicating that the degree of IR increased with age, which was consistent with the findings of previous studies [39] and which may be attributed to physiological decline, increased adipose tissue, decreased skeletal muscle, and a lack of effective interventions. Compared with Class 1 (persistently high group), the risk of new-onset CVD was significantly greater in Class 2 (significantly decreased group) and Class 3 (persistently low group). When further classified on the basis of the quartiles of cumeGDR, participants with the lowest cumeGDR had the highest risk of new-onset CVD. After adjusting for potential confounding factors, the above associations remained robust. The RCS curve revealed a negative linear relationship between cumeGDR and new-onset CVD. These findings indicate that eGDR level are independently associated with new-onset CVD.

From a mechanistic perspective, the eGDR, as an alternative marker for IR, not only reflects reduced insulin sensitivity but also captures multidimensional dysregulation, including glucose and lipid metabolism disorders, endothelial dysfunction, chronic inflammation, and oxidative stress [42–44]. These interrelated pathological processes collectively contribute to the onset and progression of CVD in patients with CKM syndrome. First, IR increases the production of very low-density lipoprotein (VLDL), and its metabolic product, residual lipoprotein, is deposited in the vascular endothelium, accelerating plaque formation [45]. Elevated free fatty acids (FFAs) in the IR state lead to increased ceramide and diacylglycerol(DAG), inhibiting glucose transporter-4 (GLUT-4) membrane translocation in skeletal muscle and adipose tissue, interfering with glucose uptake, and forming a vicious cycle of hyperglycemia and hyperinsulinemia [46]. The persistent lipotoxic environment leads to abnormal accumulation of FFAs in cardiomyocytes, directly damaging myocardial contractile function by inhibiting mitochondrial β-oxidation and increasing reactive oxygen species (ROS) production [46]. Second, the core damage caused by IR to the vascular system lies in disrupting the dual-pathway balance of insulin signaling, leading to endothelial dysfunction [47]. The PI3K/A-t pathway mediated by insulin for vasodilation is inhibited, leading to decreased endothelial nitric oxide synthase (eNOS) activity and reduced NO production, whereas the MAPK pathway is overly activated, resulting in increased endothelin-1 (ET-1), causing sustained vasoconstriction and endothelial dysfunction [47]. Notably, this process synergistically exacerbates endothelial damage with lipotoxicity. A reduction in NO weakens the antioxidant capacity of blood vessels, whereas FFA deposition directly damages the endothelium. The combined effects of these two factors lay an important pathological foundation for atherosclerosis [48, 49]. Finally, recent studies have shown that IR and chronic inflammation have a bidirectional promoting relationship, accelerating the progression of CKM syndrome [50]. IR triggers the release of FFAs and proinflammatory adipokines (such as leptin and resistin) from adipose tissue, activates monocyte differentiation into M1 macrophages, and upregulates the expression of proinflammatory factors such as TNF-α, IL-6, and IL-12 [50, 51]. This systemic chronic inflammation not only directly damages the vascular endothelium but also further inhibits tyrosine phosphorylation of insulin receptor substrate (IRS) by activating the classic IKKβ-NF-κB molecular pathway, exacerbating insulin resistance [50, 51]. Moreover, a hyperglycemic environment promotes the accumulation of advanced glycation end products (AGEs) [52] and activates NADPH oxidase, leading to excessive production of reactive oxygen species (ROS). ROS play multiple destructive roles in the pathogenesis of CVD. ROS directly oxidize low-density lipoprotein (LDL) to form ox-LDL, promote foam cell formation, and increase plaque instability [53]; they also impair myocardial cell mitochondrial function and reduce energy production efficiency [54, 55]. ROS activate the NLRP3 inflammasome, promoting the maturation and release of proinflammatory factors such as IL-1; at the same time, inflammatory cell infiltration generates more ROS. It is worth noting that the increased prevalence of CKM syndrome during the COVID**–**19 pandemic may be associated with systemic inflammatory state and metabolic disorders exacerbated by viral infection [56], further highlighting the importance of managing insulin resistance and metabolic health in the context of chronic inflammation. These multiple interrelated mechanisms collectively lead to early atherosclerosis in patients with CKM syndrome. Our findings indicate that dynamic IR monitoring can comprehensively reflect the pathophysiological processes in patients with CKM syndrome and that the cumulative effects of these pathophysiological processes are reflected in the cumeGDR. Persistently low eGDR level indicate long-term exposure to metabolic disorders, chronic inflammation, and oxidative stress, all of which synergistically increase the risk of CVD in patients with CKM syndrome. Therefore, dynamic monitoring of eGDR changes can be used to assess patients' metabolic status comprehensively and is helpful for the early identification of high-risk populations for CVD.

Furthermore, the influence of maternal nutritional status on offspring CKM health cannot be ignored. Tain and Hsu indicate that abnormal maternal amino acid metabolism can increase the susceptibility of offspring to CKM syndrome in adulthood through fetal programming effects [57]. Animal studies have shown that supplementation with arginine, citrulline, taurine, cysteine, glycine, branched-chain amino acids, and tryptophan during pregnancy and/or lactation can improve CKM-related phenotypes across multiple models [57]. Extensive animal research indicates that maternal supplementation with polyphenols (particularly resveratrol) during pregnancy and lactation constitutes an effective reprogramming strategy to prevent CKM syndrome in offspring programmed by adverse early-life environments [58]. Although our study focuses on changes in eGDR during adulthood, the developmental origins hypothesis suggests that the insulin sensitivity trajectory of an individual in adulthood may have been partially reshaped in the early stages of life. Therefore, it is imperative to advance the CKM prevention window to the very beginning of life.

Our study has several advantages, including the use of a large, nationally representative CHARLS cohort and the construction of a dynamic change trajectory of eGDR through K-means clustering, which overcomes the limitations of traditional single measurements and accurately identifies the high-risk population of metabolic deterioration in patients with stages 0–3 CKM syndrome. However, our study has certain limitations. First, only two blood tests were performed, which prevented a more detailed characterization of eGDR. When eGDR is only evaluated at a specific time, its continuous changes over time are not fully captured. Future studies with more frequent measurements could provide deeper insights into the dynamic nature of metabolic disorders. Second, although we adjusted for multiple confounding factors, residual confounding factors, such as dietary patterns and socioeconomic factors, cannot be completely ruled out and may affect the causal inference between eGDR and CVD. Our findings are particularly applicable to middle-aged and elderly populations in China and do not cover young CKM syndrome patients whose metabolic characteristics and dynamic changes in eGDR may present different patterns. The diagnosis of cardiovascular disease is based on self-reported physician diagnoses rather than imaging (CT or MRI), which introduces potential misclassification bias. However, the reliability of self-reported cardiovascular events has been validated, providing some assurance of reliability. Finally, our study adopted strict exclusion criteria (incomplete CKM syndrome data or missing CVD records), which were consistent with previous studies. Excluding certain participants may not cause bias in the study results.

As a comprehensive indicator reflecting insulin resistance and metabolic function, the eGDR can be calculated with only routine clinical data. It is low-cost, easily accessible, and suitable for grassroots settings such as community health centers and rural clinics. This study demonstrated that its dynamic changes (such as persistently low level and significant decreases) can significantly increase the risk of CVD, which is particularly applicable to primary medical institutions with limited health care resources and can be used as a tool for the initial screening of high-risk populations. Dynamic changes in eGDR can occur earlier than clinical symptoms can, suggesting deterioration of metabolic function. Early interventions such as lifestyle modification and intensive management of blood glucose/blood pressure in patients with persistently low level or significantly declining level may halt the progression of CVD and avoid the high-cost burden of late-stage treatment. The cumeGDR integrates long-term metabolic status and is more predictive than a single measurement. Its linear association with CVD simplifies risk assessment models. Clinicians can intuitively determine the long-term risk level of patients by periodically calculating the cumeGDR.

Building on the findings and limitations of this study, future research could focus on the following directions: (1) First, it is necessary to validate the generalizability and predictive value of eGDR change and cumeGDR in a wider range of populations, such as those of different races and age groups. Moreover, the integration of multiomics data, such as genomics and metabolomics data, to analyze the molecular mechanisms behind the dynamic changes in eGDR will provide key insights into understanding its biological basis. (2) On this basis, a CVD risk prediction model should be developed and validated, integrating eGDR and other relevant factors to improve the identification of high-risk individuals. (3) Ultimately, it is critical to conduct prospective interventional studies to validate whether increasing eGDR levels through specific strategies, such as lifestyle interventions for weight loss, targeted drug therapies, or nutritional supplementation approaches like Flexovital, are effective in reducing the risk of subsequent CVD events, thereby providing direct causal evidence for the development of prevention strategies, especially in the context of the growing burden of CKM syndrome.

## Conclusion

Poorly controlled eGDR level significantly increase the risk of new-onset CVD in patients with CKM syndrome stages 0–3. Dynamic monitoring of eGDR changes can optimize the risk stratification of CVD, and these findings help to more accurately identify high-risk populations and provide key evidence for early intervention to reduce the burden of cardiovascular disease.

## Supplementary Information

Additional file 1

## Data Availability

The datasets used and/or analysed during this study are available in the China Health and Retirement Longitudinal Study repository [http://charls.pku.edu.cn].

## Abbreviations

CKM: Cardiovascular-kidney-metabolic
CVD: Cardiovascular diseases
eGDR: Estimated glucose disposal rate
IR: Insulin resistance
CHARLS: China health and retirement longitudinal study
CKD: Chronic kidney disease
AHA: American heart association
HIEC: Hyperinsulinemic-euglycemic clamp
HOMA-IR: Homeostasis model assessment of insulin resistance
HbA1c: Glycated hemoglobin A1c
WC: Waist circumference
FBG: Fasting blood glucose
HPLC: High performance liquid chromatography
STROBE: Strengthening the reporting of observational studies in epidemiology
HT: Hypertension
PREVENT: Predicting risk of CVD events
SBP: Systolic blood pressure
DBP: Diastolic blood pressure
BMI: Body mass index
Scr: Serum creatinine
BUN: Blood urea nitrogen
UA: Uric acid
TC: Total cholesterol
TG: Triglyceride
HDL-C: High-density lipoprotein cholesterol
LDL-C: Low-density lipoprotein cholesterol
CKD-EPI: Chronic kidney disease epidemiology collaboration
MICE: Multiple interpolation with chained equations
SD: Standard deviation
IQR: Interquartile range
OR: Odds ratios
CI: Confidence interval
Q: Quartiles
VIF: Variance inflation factor
RCS: Restricted cubic spline
HR: Hazard ratio
TyG: Triglyceride-glucose
GLP-1RA: Glucagon-like peptide-1 receptor agonist
VLDL: Very low-density lipoprotein
FFAs: Free fatty acids
DAG: Diacylglycerol
GLUT-4: Glucose transporter-4
ROS: Reactive oxygen species
KDIGO: Kidney disease improvement global outcomes
PI3K-Akt: Phosphatidylinositol kinase/protein kinase B
eNOS: Endothelial nitric oxide synthase
MAPK: Mitogen-activated protein kinase
ET-1: Endothelin-1
TNF-α: Tumor Necrosis Factor-α
IL: Interleukin
IRS: Insulin receptor substrate
NF-κB: Nuclear factor-kappa B
AGEs: Advanced glycation end products
NADPH: Nicotinamide adenine dinucleotide phosphate
NLRP: NOD-like receptor thermal protein domain assiciated protein
