# Supplementary material for: Changes in the estimated glucose disposal rate and incident cardiovascular disease in patients with cardiovascular–kidney–metabolic syndrome stages 0–3: a prospective cohort study in China
Source: BMC Cardiovasc Disord. 2025 Nov 26;25:889. doi: 10.1186/s12872-025-05300-8 (PMC12751650; doi:10.1186/s12872-025-05300-8)
Supplement: Supplementary file 1 — Additional file 1 [file 12872_2025_5300_MOESM1_ESM.docx]

**Table S1** Distribution of variables with missing data

| **Variables** | **Number of Missi** | **Missing proportio** |
| --- | --- | --- |
| SBP | 4 | 0.14% |
| DBP | 4 | 0.14% |
| Smoking status | 4 | 0.14% |
| Drinking status | 3 | 0.10% |
| Dyslipidemia | 42 | 1.47% |
| Diabetes | 3 | 0.10% |
| Liver disease | 6 | 0.21% |
| Lipid-lowering treatment | 43 | 1.50% |
| Hypoglycemic treatment | 3 | 0.10% |
| LDL-c | 4 | 0.14% |

SBP, systolic blood pressure; DBP, diastolic blood pressure; LDL-c, low-density lipoprotein cholesterol

This ﬁle has been converted from its original format for security purposes. Please use C704681C14978 as a reference .
